# Mr. Charles Thomas Haden's Practical Observations on the Colchicum Autumnale

**Published:** 1821-06-01

**Authors:** 


*Practical Observations on the Colchicum Autumnale, as a general Remedy of great Power, in the Treatment of Inflammatory Diseases, both Acute and Chronic; and therefore as a Substitute for Bleeding in Disorders which are connected with increased Action of the Heart and Arteries.* By Charles Thomas Haden, &c. &c. &c. Burgess and Hill, pp. 84.

Colchicum would appear to comprise the virtues of the lancet, cathartics, diuretics, specifics, sudorifics, tonics, aromatics, sedatives, and expectorants; in fact, just as the alderman's beef, in the Tale of a Tub, comprised the quintessence of pease, partridges, pheasants, and quails. It was used centuries ago in catarrhs, dropsies, fevers, bronchial affections, &c. and given up for want of correct therapeutical rather than botanical knowledge. Single investigations produce perfection of experimental skill in the use of a medicine, and insight into separate qualities, as far as they can be elicited by art. By disregarding coagulability and exclusion of common air, the French brought transfusion of blood into disgrace; but Dr. Blundell has restored it to notice by his accuracy. Colchicum is similarly resuscitated, and the Messrs. Haden have done all in their power to add effect and extent to its use. Mr. H. sen. is the Mentor, "giving the multiplied experience of an old practitioner not unskilled to judge;" and Mr. H. jun. self-confessedly "non æquis passibus sequitur patrem." Ere we enter into analysis, let us just say, that we shall occasionally break in upon the arrangement in our way, in order to make our foreshortening as replete as possible with the pith of the perspective before us.

Mr. Haden, sen. first administered the various preparations of colchicum, but he found them late, and then often violent, in effect; this was ameliorated by uniting cathartics, but not effectually till it was given in the simplest form; the powder of the dry bulbs—in conjunction with a neutral salt—usually the sal polychrest. Thus he has employed it for more than six years "as his common prescription in gout, rheumatism," and "all diseases of excitement." It controls the action of the heart and arteries; is a "material auxiliary to the lancet," in diseases of excitement, and it seems to diminish action without causing any inanition of physical power; so that perfect health, rather than convalescence, demanding tonics, follows. The pulse becomes natural, and the general effect of the medicine is almost always established in pure inflammations, after the purging has taken place, and even before. Bleeding is generally superseded, unless in affections of vital parts, and then is commonly required but once. Relapses are usually slight, and stand in need of slight repetitions of the colchicum. In acute cases not relieved in twelve or fifteen hours, increased doses, with calomel or the black dose, are given to accelerate the purging. Continuation is necessary, if inflammatory symptoms remain; but if prescribed at the dawning of diseased action, this will seldom be expedient. Mr. Haden's (sen.) posological rules are, to give from 2 to 8 gr. pulv. colchic.—sulph. potass. ℈j. (vel magis) in rose mixture, every four or six hours, under the general restrictions laid down. In chronic cases a daily morning dose is given (colchic. gr. v. sulph. potass. ℈j.) in a full draught of warm water, continuing it, if necessary, for weeks, with any purgative adjunct required. (P. 17.) Bleeding postponed is sometimes superseded, says Mr. Charles Haden, especially in inflammatory fevers, pneumonia, &c. On the contrary, though it may succeed by itself, in one case of pneumonia or inflammatory rheumatism, it may merely act as auxiliary, with active and repeated blood-letting, in another. A case is given which expresses an important diagnostic remark, that invigorating diet and medicine produces relapse in the convalescent stage of sthenic diseases with an unclean tongue. By its different appearances the author hopes, in time, to detect the primary situations of irritative and inflammatory action. In another description of irritative and inflammatory action, a learned friend of ours has surprised us by the accuracy with which he distinguishes the mucous from genuine phthisis, by the peculiar streak in the middle of the dorsum of the tongue, and its furred edges. A quick pulse certainly commands caution, but who has not seen the tongue furred from sheer debility, and become rubrous as a cherry on moderate invigorating diet, &c.? Sudden shocks to the nervous system produce a furred tongue: we have a case of gun-shot wound of the thorax, where it was formed an hour after the accident—before any particular disturbance of the constitution had followed.*

* Mr. C. Haden gives a case of acute rheumatism, reduced by Colchicum and bloodletting, but which afterwards rallied from the premature use of bark, the tongue being unclean. May not the former drug render the symptoms passive by its sedative properties, whilst the disease itself may actually continue to exist? All remedies speedy in their action generally require to be proportionally protracted—or it will be found that the seeming effect is rarely real.

Mr. Charles Haden's mode of exhibition and rules for the limitation of the remedy do not materially differ from his father's. His officinal preparation is composed of one part of powdered colch. 3 carb. of potass, and 5 sulph. potass. The dose is ʒj. three or four times a day, with half a pint of warm water, in effervescence with tartaric or citric acid. After purging, if likely to be ill borne, the colchicum may be given alone. In chronic complaints calomel or blue pill, and aloes with ipecacuan, form a useful night pill, with one or two dram doses of the compound daily. The childrens' dose of the same varies from 16 gr. to ℈ij. Mr. H. sen. says,—

"In organic derangements of structure, *when occasionally* attended by inflammatory symptoms, the above treatment answers perfectly in curing *super-induced* inflammation; so much so, that at times the *general actions* are so much subdued as to give no notice, by symptoms, of the existence of the primary diseases; *thus*, in one case, gangrene, was going on in the foot, whilst the inflammation which produced it was subsiding under the use of colchicum."

Colch. gr. vj. sulph. potass. ʒj. *omni mane* has never failed with long perseverance in chronic rheumatism. Plethoric hæmorrhages passive or habitual, and consumptions, (though "it does not cure,") are relieved without the expense of blood, and the severe consequences of accidents are averted by it.

The elder Mr. Haden's horse had a violent inflammatory attack of the chest and joints, which yielded *too* effectually to doses of colch. ʒij. magnes. sulph. ℥j. $6^{tis.}$ horis, but the animal was much purged, and had colic—which is attributed to overdosing, and neglect of spice in the equine prescription. We have seen fatal colic follow the administration of a cold clyster to a horse.

"In the fevers of children in which colchicum generally succeeds, opening medicine is often an indispensable requisite in the prescription."

In the case of a child nothing availed till a decided purgative brought away a load of fæces. In nine instances out of ten this case will represent the just treatment of complaints of children, and colchicum will, in our opinions, have nothing at all to do with it. Indeed, throughout the whole of these cases, we cannot help suspecting that the secondary component is not raised to its just level; but Mr. Haden, however, admits throughout the indispensable necessity of combined purging. In lumbago great stress is laid upon using warm bathing, and warm beverages in conjunction; sometimes calomel and antimony is recommended,

Another case multifariously treated finally appeared to yield to prussic acid. A case, after three weeks mal-treatment, yielded to one dose of colchicum. Mr. Haden, sen. reports one affecting all the limbs.

"On the day but one after taking (colchicum) he (the patient) was met walking in the street, and was very soon well. Five doses of the medicine were taken."

Some interesting cases of bronchitis, in the worst form, exhibit its utility, as first stated by Dr. Hastings, and also recommended by Dr. Abercrombie,* who uses it in combination with super-tartrite of potass.† Infantile pneumonia, inflamed breasts and nipples, with constitutional irritation, and simple fever, are among the cases detailed as successfully treated. Mr. Haden, jun. contributes these cases, it being stated in the early part of the work that Mr. H. sen. laid the foundation for these extended trials by giving it invariably in rheumatic inflammatory fevers, catarrhs, influenza, and fevers of puerperal patients, in which last it "has acted like magic." A severe case of mixed bronchitis and pleurisy yielded on the third day to colchicum, except that the expectoration and cough continued, which relapsed on making an exchange for tartar emetic and opium, and yielded finally to colchicum resumed, with calomel and V. S.

Colchicum, when disagreeing, excites languor and sickness, but this is not peculiar; squills and antimony produce the same *loathing* nausea; but having caused this effect by digitalis and vin. antim. we have once or oftener substituted colchicum, without re-exciting nausea, as might be expected, the stomach being first reconciled by gentle alcoholic stimuli. Colchicum, with us, has just proved useful in a case of inflammatory rheumatism of the thorax, but it now disagrees in the foregoing manner. In cases of gout with hysteria in the female, we have found it also generally obnoxious to the stomach.

Having mentioned Mr. Haden's decisive cases, we shall briefly notice the section which comprises the "subacute and chronic."

"*On the same principle* the cutaneous diseases which so abound among the patients who apply for relief at the Chelsea and Brompton

---

* Pathology of Consumptive Diseases, *Edinb. Med. Journal,* LXVI. *Jan.* 1821.

† See Rev. on the Mucous Membrane of the Thorax, *Art.* I. *No.* 3. *Med. Chir. Rev.*

Dispensary, are more benefited by the use of colchicum than any other remedy." 87.

A very impressive case is given in confirmation, consisting in porrigo favosa, with constitutional affection. When the disease is purely cutaneous, Mr. Haden does not observe its powers to be exerted so successfully; this is just what the pathology of cuticular affections would lead us to expect. In nine cases out of ten we believe this form of disease to be a salutary exchange of diseased actions with some important viscus. Whoever shall have read Dr. Willan's entertaining, but too costly, quartos, will find this proposition remarkably verified by statements, though little taken into account; and whoever has an eye and a mind for tracing up the first phenomena of morbid actions, will be the more convinced of this important physiological truth. If one internal organ more than another is connected as a primary link in the chain, we believe it to be the liver. Some fœtal dissections which we have lately made, have induced us to entertain an opinion that many parts of our organization, colour of the skin, its texture, the characteristics of the hair, and temperament itself, are somewhat connected with the early development of this great gland and its economy; there may be also a reciprocal dominion of the brain in this early connexion. We hazard this as mere conjecture. If it be too wild, we err with the ancient philosophers, who grounded their temperamental distinctions much (too much perhaps) upon the qualities and supposed various conditions of the bile. The first and more substantial theme of these remarks, relating to the connexion between establishments made on the skin for guarding masked derangements of internal organs, especially the liver, is confirmed not merely by the action of the colchicum, or still more of the taraxicon leontodon, but by the celerity with which the skin is often rendered immaculate in the worst dermalogical cases, by the use of mineral waters, as the Cheltenham spas. In a short period we hope to see a most interesting physiological and pathological treatise, conceived in these principles, and illustrative of some important laws of the animal economy and mucous system (using Bichat's phrase) from the pen of one of our first veterans. Whenever it appears, there will be much to excite attention, and perhaps applause, as one of the most original efforts of an original and matured mind.*

* We suppose our reviewer alludes to the forthcoming "Letter to Dr. Charles Parry, by Dr. Jenner, on the Influence of Factitious Eruptions." We shall hail with pleasure this long-matured production of that venerable philosopher. *Ed.*

The influence of colchicum is displayed favourably in numerous and dissimilar affections, corresponding in one point, the presence of febrile excitement. Let us solicit regard—

"To that of threatened consumption, and that of actual phthisis, as being interesting in many points of view; to the two of inflamed leg, as fully illustrating the great powers of colchicum, in remedying states of vehement local excitement; to that of tubercular cutaneous diseases; as proving its efficacy in the treatment of skin disorders, when joined with an excited state of the constitution." 69.

Let us add—to that of derangements of the digestive organs, sometimes accompanied with spontaneous eruptions; bursal inflammation, with constitutional irritation; hysteria, with excitement; cynanche tonsillaris; boils; indisposition consecutive upon infantile laryngitis, as adapted to impress the mind with comprehensive ideas of the powers of this versatile and extraordinary galenical.

Mr. Haden confidently recommends the powder as more safe and certain, in exception to tinctures of the bulb, but speaks with more satisfaction of the preparations of the seeds on Dr. Williams' authority. The sanguine expectations of the Messrs. Haden are strongly sanctioned by the reports of Dr. W.* Dr. Williams' experience of the common preparation closely accords with Mr. Haden's, in some cases being inert, in others exciting excessive action of the stomach and bowels, so that it has occasionally proved fatal. Perhaps much is attributable to not previously clearing the bowels. It it often observed by practical men that powerful medicines are uncertain and capricious without this caution; digitalis given without effect for some time, will sometimes act by a sudden burst, as in the full force of combination from the same apparent neglect. There are numerous affections in which, if the membranes and muscular coats of the alimentary tube are not first excited by purgatives, want of action and excitability may be expected after the exhibition of other stimuli. Dr. W. thinks the seeds may be extended to all painful diseases of the *asthenic* kind, and we know but of one objection—their extreme dearness and scarcity. A learned physician having the care of a large institution, has been trying the seeds, with mixed success, but less than anticipated.—There is a rheumatism connected with disordered functions of the duodenum, in which we have observed colchicum to fail.

---

* See Med. Chirurg. Review. March No. Favourable Reports. April No. Medical Repository.

Some modern as well as the ancient writers on materia medica consider colchicum inert, except in spring and July. Mr. A. T. Thompson, whose authority is great on such points, deems a blue stain, produced by rubbing colchicum with alcoholic tinct. guiaci and acetic acid, a test of its perfection. We have heard much of Mr. T.'s habits of accuracy in the nicer operations of chemistry, but we are disposed to think him in error in maintaining the reverse of Mr. Battley's proposition, that drying at a high temperature (170) extinguishes the efficacious properties of the bulb. The most powerful powder which Mr. C. Haden has met with, was gathered in September, and dried at 130°. (p. 77.) A communication addressed to us by Mr. J. Fosbroke, a young practitioner in the country, and an occasional contributor to the periodical literature of the day, concurs with this statement. We shall here introduce a short extract.

"I have of late had much practice in gout and rheumatism, with one very extraordinary case of masked gout. I am tracing up a series of minute observations on the action of colchicum, Pradier's poultice,* (which does wonders in rheumatism as well as gout) and on sulphureted hydrogen, &c. which I shall make known in due time. Finding the acetum colchici of the pharmacopœia, obtained from the summer bulb, too inert, I was obliged to betake myself to an extemporaneous preparation formed from that obtained by the digging of the root, and superintending it in all its stages myself. The officinal composition of the pharmacopœia is formed by the rad. colch. ℥j. to acid. acetic. Oj. whereas the vinum colchici is in the proportions of wine ℥xxiv. to bulbs ℔ij. a disparity which seems inconsistent. From this opinion and disappointment in the effect of the pharmacopœial preparation, I macerated at the beginning of October ℔ij. of the fresh bulbs in three pints of common vinegar for a fortnight, and then added sp. vin. rect. ℥i. to preserve it. This was administered in the dose of a drachm to a femaleg outy patient. The kidneys, which were previously in a torpid state, yielded in one night a gallon of urine; it also acted on the bowels several times, produced some nausea, perspiration, and uneasiness of the stomach and intestines. I have since been obliged to give it in very small doses, as it continued to evince full powers. This experiment seems to jar with the opinion that the root is acrimonious, and endowed with its sensible qualities as a drug *only* at midsummer, and not in the autumn, when I took it up. According to my observation, successive crops spring up from July to October, the first crop being more ample than the latter, which is longer in attaining maturity. The tunics were full at this time of insterstitial fluid, extremely pungent in smell. I prefer the acetic preparation, because, as Dr. Scu-

* Essay on the Treatment of Gout in all its Forms. By James Johnson, M. D. &c.

damore observes, it may be administered in the simplest form by neutralizing the acid with an alkali or alkaline earth.* Thus given, it loses much of that flavour and odour to which the sensitive and feeble stomach is very repugnant. It appears to me, in one or more cases in combination with magnesia or Epsom salts, to have been as periodical in its purgative action as aloes. Dr. Parisre marks, that if diuretics are given in purgative doses, they fail to excite diuresis, which seems true, and points out caution in the exhibition. An eminent general practitioner in the country informs me that in a case of dropsical effusion, where other remedies had failed, he had obtained a rapid cure by the administration of colchicum. My use of it has been confined to cases of gout and rheumatism. Previous to maceration I cut the bulb into thin transverse slices, and dried them by a moderate heat."

It remains for us to state our own experience, and that of some of our friends. It has been our habit to give the acetous preparation, as directed in Dr. Scudamore's history and treatment of gout, modified in combination, as suited particular circumstances. In gout we have not once failed, in a considerable number of cases. In one more obstinate than the rest, where the effects of an overdose were complained of, the disease was considerably mitigated. When suspended, on similar account, in other cases, good effects have appeared after tranquillity has been restored to the organs of digestion and secretion. We have experienced its beneficial effects when combined with gentle laxatives to act mildly, surely, and equally, upon the intestinal canal, liver, kidneys, and skin. To effect this comprehensive influence in constitutions of much diversity, we have been obliged to vary the combinations as circumstances required. If diuresis demanded to be pushed, we have added digitalis or elaterium; in obstruction of the biliary system, the liver may be expeditiously relieved through the medium of the kidneys, by adding decoction of dandelion, a medicine to which we never found a patient unwilling to recur. In spasmodic cramps, and irregular nervous spasms, the stomach may be guarded from aversion to colchicum by the union of sulphuret of potass. As a purgative in gout, the decoct. aloes co. which neutralizes the acetum colchici when given with it, is eminently useful.† Thus administered, the acetum is almost

---

* On Gout, 3d Edition, p. 186.

† In adult cases, where the mucous membrane of the lungs has been embarrassed by vitiated mucus giving rise to a spurious asthma of the spasmodic kind, while at the same time the lining coat of the alimentary canal has been similarly loaded, causing singular anomalous and sympathetic affections of remote muscular structures, and pains of the joints, equivalent in simplicity to the powder. As medical writers often catch the water instead of the fish, in reasoning on the operation of medicines, we shall speak of the *modus operandi* of colchicum with caution. We attribute much to its equal and divided effects upon the organs of secretion, when in combination—much to the simultaneous influence of these manifold qualities—much to its tendency to relieve plethoric circulation in the veins; and we value it for diminishing nervous and arterial energy, so little at the expense of muscular power. It is no depreciation of the value of colchicum to say that we have no unequivocal proofs of its efficacy when singly exhibited; the art of combination is too seldom well managed, and the judicious use of every medicine greatly consists in thus creating and varying the effect. In Mr. C. Haden's cases, we do presume that he has given its powers a rather unnatural direction; and indeed he admits the too liberal addition of purgatives, and thinks that his practice may have been consequently less efficacious than his father's. Perhaps it is, Dr. Paris says, too much diverted from the kidneys. The fundamental principle on which Dr. Scudamore has founded his treatment of gout, was derived from the early reports of the action of the eau medicinale, viz. simultaneous excitement of all the secretions.* We have means of stating, from a relation of Dr. Huisson, that the basis of this celebrated specific is a concentrated preparation of colchicum and briony. This or any other *uncombined* preparation of colchicum will always probably be injurious in most affections, but we are sorry that a medicine possessing such powers should have undergone no trials in modification. In the case of an individual of the sanguine temperament, with hereditary gout, the eau medicinale produced, to our surprize, that singular black exudation of the gouty joints, which has been observed upon Pradier's poultices, or its substitutes. To continue speaking of colchicum in its general bearings, a medical friend informs us, "I have been using the vin. colchic. in inflammatory rheumatism translated from the wrists to the pericardium and pleura. Its immediate operation was cathartic and emetic, and the patient suddenly recovered as far as the chest was concerned. The disease returned in a

---

we have found nothing that exceeds the acet. colchici neutralized with magnesia, and combined with tartar emetic, for removing the *irritative* action of the mucous membranes, by restoring the languid action of the skin, and dislodging, by expectoration and purging, viscid mucus and slime from the bowels and trachea.

* See article Gout, in Rees's Cyclopœdia.

mitigated form in his extremities, and hung partially about him for some time. The patient being at the brink of the grave, I prescribed the colchicum with scarce a ray of hope. In chronic rheumatism it generally does good. I have now an athletic patient, 23 years of age, who has had acute rheumatism in the knees and arms; it left these parts, and his chest became affected; the pericardium most prominently. Copious v. s. and vin. colchici. He is progressively improving." We ourselves have recently been using vin. colchici in metastatic rheumatism of the pleura and pericardium without effect in ʒj. to ʒij. doses. In this dreadful case of two months standing, there have been six general blood-lettings, at very short intervals, blistering, purging, sinapisms, digitalis, leeching to syncope repeatedly, with slight mitigations only. The patient is now living, but circumstances have prevented our using the powders, or further attending to the case. We have lately checked the progress and mitigated the pain of an abscess deeply seated in the shoulder of a rheumatic patient by the acet. colchici. In exhibiting it according to Mr. Haden's formulæ, in cases of biliary obstruction, not mechanical, in hysteria, with vascular irritability, acute inflammation of the mucous membranes of the intestines, apoplectic determinations, we have by no means found it infallible, or to be confided in alone. An extreme thirst, with a subjection of active symptoms for a time, not unlike the effect produced through the medium of the brain and nervous system by that class of medicines called sedatives, has followed in many instances. In children's cases, as urticaria with intestinal disorders, and similar affections, we find it very efficacious. Professor Herberski, in the chair of materia medica at the university of Wilna, has personally informed us, that in Germany and Poland it has been long used and overrated, but that it is considered to possess considerable influence in emulging the biliary and urinary glands.

We have been so closely busied on the valuable parts of Mr. Haden's work, (and as a collection of facts there is very much that is valulable) that we have been sparing of criticism. We ought to have remarked some incongruities in the application of the proofs to the propositions; but above all, we consider his general arrangement unconformable to any law with which we are acquainted, except the laws of writing in the order of a day book, from which his work appears to be a transcript.

We conceive that in his title page Mr. Haden has promised too much—particularly as to the favourite medicine being a substitute for bleeding in inflammatory complaints

—a position contradicted, we imagine, by some of his own facts. These trifling strictures, however, we are confident the author will take in good part, like a man of sense and science, who can distinguish between wanton criticism and candid commentary.

---

